# Empyema caused by *Fusobacterium nucleatum* with squamous cell carcinoma of the lung: a case report and literature review

**DOI:** 10.3389/fmed.2023.1099040

**Published:** 2023-05-25

**Authors:** Yue Sun, Han Dong, Na Zhang, Peng Zhao, Yuan Qi, Xin Yang, Lingling Wang

**Affiliations:** Department of Respiratory and Critical Care Medicine, The Fourth People's Hospital of Shenyang, Shenyang, China

**Keywords:** *Fusobacterium nucleatum*, empyema, next-generation sequencing, squamous cell carcinoma, lung cancer

## Abstract

**Background:**

*Fusobacterium nucleatum* is a common oral symbiotic flora that can cause respiratory tract, oral nervous system, obstetric and skin infections. *Fusobacterium nucleatum* infections are mostly caused by aspiration. The clinical manifestations of pulmonary infections with *Fusobacterium nucleatum* can include simple pneumonia, lung abscesses, empyema, etc.

**Case presentation:**

We described the case of a 49-year-old man with a 1-year history of intermittent cough and sputum production who had worsened over the last 4 days with fever and right chest pain. After thoracentesis and catheter drainage were performed, *Fusobacterium nucleatum* was detected in the pleural effusion by using next-generation sequencing. Meanwhile, a diagnosis of squamous cell carcinoma of the right lung was made by fiberoptic bronchoscopy. The patient's condition improved significantly after percutaneous drainage and long-term intravenous antibiotic treatment.

**Conclusions:**

This is the first case reported of empyema due to *Fusobacterium nucleatum* infection in a patient with squamous cell carcinoma.

## Introduction

Empyema is a suppurative infection caused by pathogenic bacteria invading the pleural cavity and produces purulent exudate that accumulates in the pleural cavity. Risk factors associated with empyema include bacterial pneumonia, pulmonary abscess, mediastinal infection, damage to the chest wall or esophagus, bacteremia or sepsis, pleural puncture, iatrogenic infection, etc. (1). A study evaluated 198 patients with pleural empyema, 74.2% of whom tested positive for anaerobic bacteria. *Fusobacterium nucleatum* was detected in 27.2% of the samples that grew anaerobes (2). Due to the difficulty in the isolation and cultivation of anaerobic bacteria in clinical work, the actual detection rate of anaerobic bacteria is low. *Fusobacterium nucleatum* is a common oral symbiotic flora that is also found in the digestive and genitourinary tracts. *Fusobacterium nucleatum* has also been linked to the reactivation of colorectal adenocarcinoma and inflammatory bowel disease. In fact, the reported incidence of Fusobacterium infections is between 0.6 and 3.5 cases per 1 million population (3).

Here, we report a case of squamous cell carcinoma of the right lung with empyema that was diagnosed by fiberoptic bronchoscopy, and next-generation sequencing was used (NGS) for the detection of a Fusobacterium infection in the pleural effusion.

## Case report

We described the case of a 49-year-old man with a 1-year history of intermittent cough and sputum production who had worsened over the last 4 days with fever and right chest pain. The patient recently had a history of unexplained weight loss of 5 kg. He has smoked 20 cigarettes per day since the age of 19. The patient had periodontitis and tooth loss. The patient had no history of travel or tuberculosis exposure and no food or drug allergies.

On admission, his temperature was 37.9°C, pulse rate 81 beats/min, respiratory rate 22 breaths/min, blood pressure 132/80 mmHg, and oxygen saturation 95% on room air.

Pulmonary percussion found dullness in the right lower lung, and pulmonary auscultation revealed decreased breath sounds in the right lower lung. The remaining physical examination was normal.

**Figure 1:**
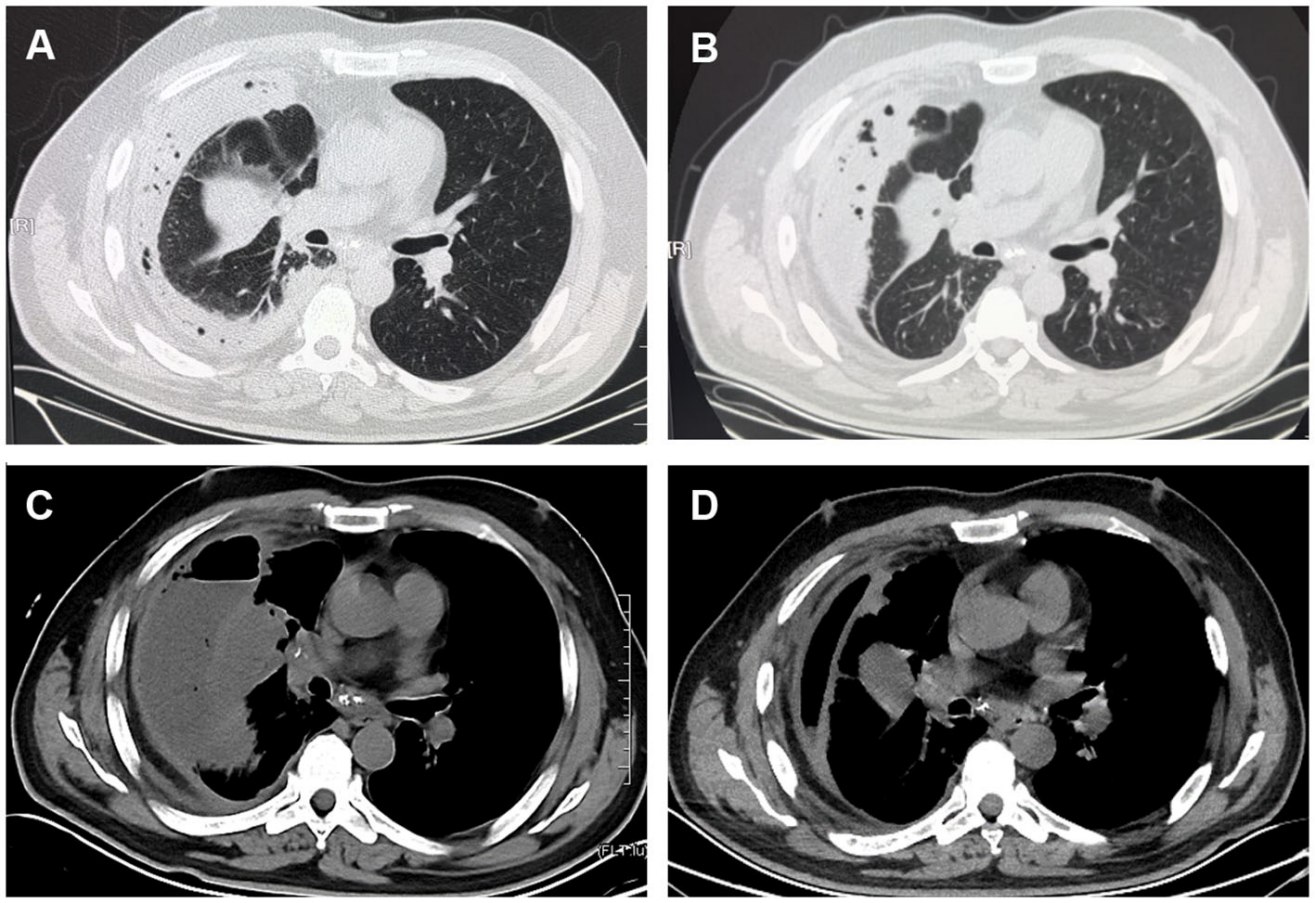
CT scan images. **(A)** Chest CT showed an irregular mass at the right middle lobe of the lung near the interlobar fissure with right atelectasis and encapsulated effusion and pneumatosis. **(B)** Chest CT showed no significant changes in the amount of right-sided pleural effusion after thorough pleural drainage and anti-infective treatment for 3 weeks. **(C)** Contrast-enhanced CT scan showed uneven enhancement of the mass in the lateral segment of right middle lobe, mediastinal lymphadenopathy with visible enhancement, and a right hydropneumothorax on April 1. **(D)** Two weeks after readmission, chest CT showed that the right-sided pleural effusion and gas were significantly reduced.

The laboratory findings were as follows: white blood cell (WBC) count 22.98 × 10^9^/L with 85.5% segmented neutrophils, 7.6% lymphocytes, neutrophil count 19.9 × 10^9^/L, and lymphocyte count 1.76 × 10^9^/L, and his ultrasensitive C-reactive protein (hs-CRP) was 248.56 mg/L (normal range 0–5 mg/L). Serum tumor marker screening showed a slightly elevated neuron-specific enolase (NSE) of 17.05 ng/ml (normal range <16.3 ng/ml). The squamous cell carcinoma antigen (SCCA), cytokeratin-19-fragment (CYFRA21-1) and carcinoembryonic antigen (CEA) levels were normal. The other laboratory test results were normal. Chest computed tomography (CT) revealed an irregular mass in the right middle lobe of the lung near the interlobar fissure with right atelectasis and thickening of the right pleura with encapsulated effusion and pneumatosis (Figure 1A). Thoracentesis and catheter drainage were performed immediately after chest ultrasound confirmed pleural effusion. Analysis of the drained purulent fluid suggested an exudative process [fluid protein 54.9 g/L, serum protein 69.8 g/L (normal: 65–85 g/L), fluid lactate dehydrogenase 3,014 U/L, serum lactate dehydrogenase 220 U/L (normal 120.0–250.0 U/L)]. Other pleural fluid analysis showed WBC 3,800/μL with 80% neutrophils, fluid glucose 0 mmol/L, and fluid adenosine deaminase 81.8 U/L. Pleural fluid smear, Gram staining, bacterial culture, acid-fast bacilli culture and smear, and cytology were all negative. A blood culture was also negative. Empyema was diagnosed. He received intravenous imipenem/cilastatin 1.0 g once every 8 h. After repeated pleural effusion drainage and anti-infective treatment for 3 weeks, re-examination of lung CT showed no significant change in the amount of pleural effusion (Figure 1B).

**Figure 2:**
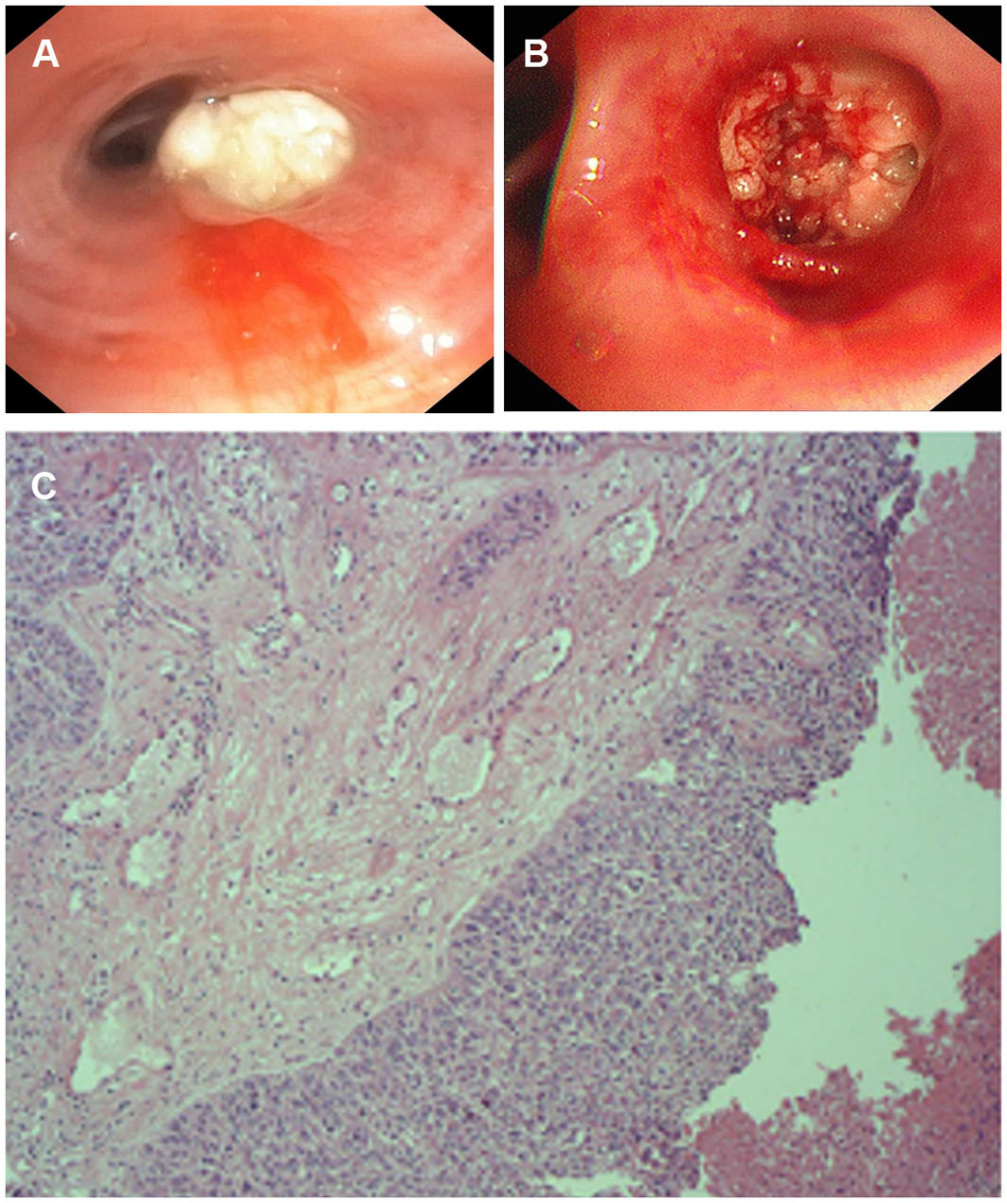
The appearance of fiberoptic bronchoscopy and Pathological examination. **(A)** Complete stenosis of the airway in the lateral segment of the right middle lobe due to white endobronchial lesions. **(B)** A cauliflower-like mass was seen and biopsied. **(C)** Pathological examination of bronchial tissue in the middle lobe of the right lung shows a high-differentiated squamous cell carcinoma.

The patient requested to be discharged after his fever improved. Half a month after discharge, he was admitted to our hospital due to progressive worsening of cough and shortness of breath. On April 1, a contrast-enhanced CT scan showed uneven enhancement of the mass in the lateral segment of the right middle lobe, mediastinal lymphadenopathy with visible enhancement, and a right hydropneumothorax (Figure 1C). In addition, the laboratory test results included WBC 11.56 × 10^9^/L, neutrophil ratio 71.84%, and CRP 219.35 mg/L. Ultrasound re-examination showed that there was a localized anechoic area of 5.3 cm × 7.7 cm × 3.4 cm in the right thoracic cavity, and he underwent pleural puncture and drainage again. The pleural drainage fluid was sent for NGS. The NGS results only detected *Fusobacterium nucleatum*. Subsequently, the patient underwent bronchoscopy. Fiberoptic bronchoscopy showed complete airway obstruction due to whitish endobronchial membranous lesions in the lateral segment of the right middle lobe (Figure 2A). A cauliflower-like mass was seen and biopsied after clamping the whitish endobronchial membranous lesions (Figure 2B). Histological examination of the biopsy specimens demonstrated a highly differentiated squamous cell carcinoma in the right lung (Figure 2C). He was finally diagnosed with empyema caused by *Fusobacterium nucleatum* and squamous cell carcinoma of the lung. The patient's cough and shortness of breath were improved after draining 1,800 ml pleural effusion for 3 days, and cefoperazone sodium/sulbactam sodium 3 g was given intravenously twice a day for 2 weeks. Repeat chest CT revealed absorption of the fluid and gas in the right pleural cavity (Figure 1D). Meanwhile, blood biochemical examination showed that infection indexes, such as white blood cells and CRP, had returned to normal. Regrettably, the patient refused antitumor therapy.

## Discussion

*Fusobacterium nucleatum* is a human anaerobic Gram-negative bacterium that colonizes the oral cavity and was originally isolated from dental plaque, and it is often closely related to the development of periodontal disease and other oral diseases (4). There are 13 species in the Fusobacteriaceae family, among which *Fusobacterium nucleatum* and Fusobacterium necrophorum are the most common invasive pathogens in humans. Lipopolysaccharide endotoxins in the outer membrane of this bacterium, which help bacteria to accumulate and invade tissues, are common pathogenic factors. In addition to the oral cavity, *Fusobacterium nucleatum* can be isolated from the human blood, genitourinary system, brain, lung, liver and other organs where abscesses can form (5). Pleuropulmonary infections caused by *Fusobacterium nucleatum* could present as simple aspiration pneumonia, necrotizing pneumonia, lung abscess (6), and empyema (7, 8), and these infections are often due to oropharyngeal aspiration in patients with periodontal disease (9). In this case, the patient had extremely poor oral hygiene with periodontitis and tooth loss, which increased the risk of aspiration of *Fusobacterium nucleatum* into the oral cavity and into the pleural cavity, resulting in empyema.

In addition, squamous cell carcinoma obstructing the right middle lobe plays an important role in pleuropulmonary infection. Lung CT (Figure 1C) showed a mass of 3.2^*^5.4 cm in the right middle lobe, which had atelectasis. A cauliflower tumor obstructing the airway could be seen after removing the white necrotic tissue. The abundant blood supply of the tumor allowed for the proliferation of *Fusobacterium nucleatum*, which could then be inhaled into the airways. *Fusobacterium nucleatum* has been shown to invade the intracellular compartments of tumor cells (10). CT examination could not determine whether the tumor in the right middle lobe was benign or malignant, but timely bronchoscopy and bronchoscopic biopsy confirmed the diagnosis. The etiology of cancer increasingly recognizes chronic infection and inflammation as components in carcinogenic feedback loops including the local microbiota. The cancer-promoting features of *Fusobacterium nucleatum* include bacterial invasion and inflammation (e.g., flagellar assembly and bacterial chemotaxis) (11–13), metabolic pathways (e.g., homolactic fermentation) (14), creation of DNA-damaging substances (15), and encouragement of cell proliferation (e.g., E-cadherin/β-catenin signaling through unique FadA adhesion) (16), suggesting the potential role of *Fusobacterium nucleatum* in the early stages of tumorigenesis. Interestingly, a comprehensive analysis found that *Fusobacterium nucleatum* enrichment in head and neck squamous cell carcinoma tissues was strongly associated with non-smokers, lower tumor stage, lower recurrence rates, and better cancer-specific survival (10), which in contrast to low survival and poor prognosis for *Fusobacterium nucleatum* infection in colorectal and esophageal cancers (17–19). The association between *Fusobacterium nucleatum* and lung cancer has not been reported. Therefore, more research is needed to explore the underlying mechanism of *Fusobacterium nucleatum* in the development of lung malignancies. In addition, This patient may develop recurrent lung infections due to poor oral health (20–22) and obstructive pneumonia complicated with lung cancer (23, 24), resulting in clinical decline and poor prognosis.

This is the first reported case of empyema secondary to a *Fusobacterium nucleatum* infection detected by NGS. The traditional detection method is bacterial culture, and the recommended medium is Brucella-based or fastidious anaerobe agar, which can improve the detection rate (25). The detection rate of anaerobic bacteria can be up to 70% by next-generation sequencing (NGS), which is better than the 20% by traditional bacterial culture technology (7). NGS combined with semiquantitative PCR was reported to be more helpful in the diagnosis of the pathogenic bacteria of pleural empyema and parapulmonary effusion (26). Compared to the difficulty of traditional culture, NGS was able to amplify and detect cell-free DNA and DNA fragments in dead cells to determine the type and number of pathogens (27). Negative results for all pleural fluid cultures, partly due to the nature of anaerobic bacteria that are difficult to culture, or partly due to limited microbiological testing techniques in hospitals (only blood agar plates can be performed). Therefore, with the patient's consent, we tested the patient's pleural fluid with NGS. This case highlights the application of NGS in the etiological diagnosis and guidance for the treatment of empyema.

## Conclusion

In conclusion, this case reported a patient with squamous cell cancer with empyema due to an infection by *Fusobacterium nucleatum*, and NGS and bronchoscopy helped to quickly identify the pathogens and pathology. Early pleural effusion drainage and a full treatment course with effective antibiotics are imperative.

## Data availability statement

The original contributions presented in the study are included in the article/supplementary material, further inquiries can be directed to the corresponding author.

## Ethics statement

Written informed consent was obtained from the patient for the publication of this case report.

## Author contributions

YS wrote the first draft. LW revised the manuscript. HD and NZ recorded the medical information. YQ, PZ, and XY contributed to the treatment of the patient. All authors contributed to the critical revision and provided final approval of the submitted version of this article.
